# Use of a Structured Method of Antimicrobial Stewardship at a City Hospital by Pharmacy Students

**DOI:** 10.3390/pharmacy8040188

**Published:** 2020-10-14

**Authors:** Kimberly E. Ng, Nicole Bradley

**Affiliations:** Department of Clinical Health Professions, College of Pharmacy and Health Sciences, St. John’s University, Queens, NY 11439, USA; ngk3@stjohns.edu

**Keywords:** pharmacy, student, antimicrobial, stewardship, tool

## Abstract

The objective of this study was to determine the utility of a structured method of antimicrobial stewardship by Advanced Pharmacy Practice Experience students and assess student perceptions of the tool. Pharmacy students on rotation were trained to utilize a structured team antibiotic review form (TARF) as a tool to participate in antimicrobial stewardship. Students completed anonymous evaluations regarding their confidence in performing stewardship after completing their rotation, and preceptors quantified total student interventions. Data analysis was conducted using descriptive statistics. The Fisher’s Exact Test was used to compare students’ confidence before and after using TARFs. Twenty-six students participated in antimicrobial stewardship using TARFs, resulting in 889 interventions. Nearly 96% of students reported that TARFs helped them evaluate patient antibiotics in a way that was easy to follow and that TARFs provided them with an organized and structured way to systematically evaluate antibiotics. All students felt that the TARFs increased their knowledge on how to evaluate antibiotics. Significantly more students were confident in participating in antimicrobial stewardship after using the TARF. TARF use allowed students to substantially contribute to stewardship, and provided them with a structured guide allowing for improved student knowledge and confidence.

## 1. Introduction

Antibiotic therapy is critical in the treatment of various infections. Prompt initiation of antibiotics has been shown to reduce morbidity and mortality, such as in cases of sepsis. However, one third of antibiotic prescriptions in United States (U.S.) hospitals are considered unnecessary or suboptimal, and may involve prescribing issues such as initiating an antibiotic without proper testing or inappropriate duration of use [1]. Antimicrobial stewardship (AS) is a coordinated set of interventions that aims to increase appropriate use and prescribing of antibiotics in healthcare settings. Antimicrobial stewardship programs (ASPs) improve patient safety by reducing the risk of serious adverse drug reactions and the development of antimicrobial resistance while increasing cure rates and correct prescribing. Pharmacists have been identified as important members of ASPs, with the Joint Commission requiring involvement of pharmacists as a standard [2].

The American Society of Health-System Pharmacists (ASHP) recognizes the current shortage of pharmacists with advanced training in infectious diseases to fill these roles and supports the expansion of pharmacy education and postgraduate training in order to develop an adequate number of pharmacists to deliver these essential services [3]. At our hospital, regular ASP participation by a pharmacist is limited and inconsistent due to staffing constraints. Currently, one pharmacist is responsible for performing stewardship, but due to the size of the hospital and quantity of antibiotic prescriptions, only select antibiotics are reviewed on a regular basis. Two pharmacy faculty and multiple pharmacy students are available to assist with stewardship activities.

Limited data on the role of pharmacy students in ASPs are available. In 2018, Childs-Kean and colleagues conducted a literature review investigating involvement of medical and pharmacy trainees in AS. Four studies involving pharmacy trainees were identified. While trainees in these studies included both introductory pharmacy practice experience (IPPE) students and advanced pharmacy practice experience (APPE) students, most trainees were postgraduate [4]. Two of the studies looked at the role of APPE students in ASP [5,6]. In the study conducted by Benson, APPE students contributed to prospective audit and feedback, dose optimization, and antibiotic de-escalation. Decreased antimicrobial costs were observed after incorporation of pharmacy students into the ASP [5]. In the study by Laible and colleagues, APPE students and PGY-1 and PGY-2 pharmacy residents contributed to prospective audit and feedback, dose optimization, and antibiotic streamlining [6]. No prior studies have detailed a structured method of AS as a tool for APPE students.

**Figure 1:**
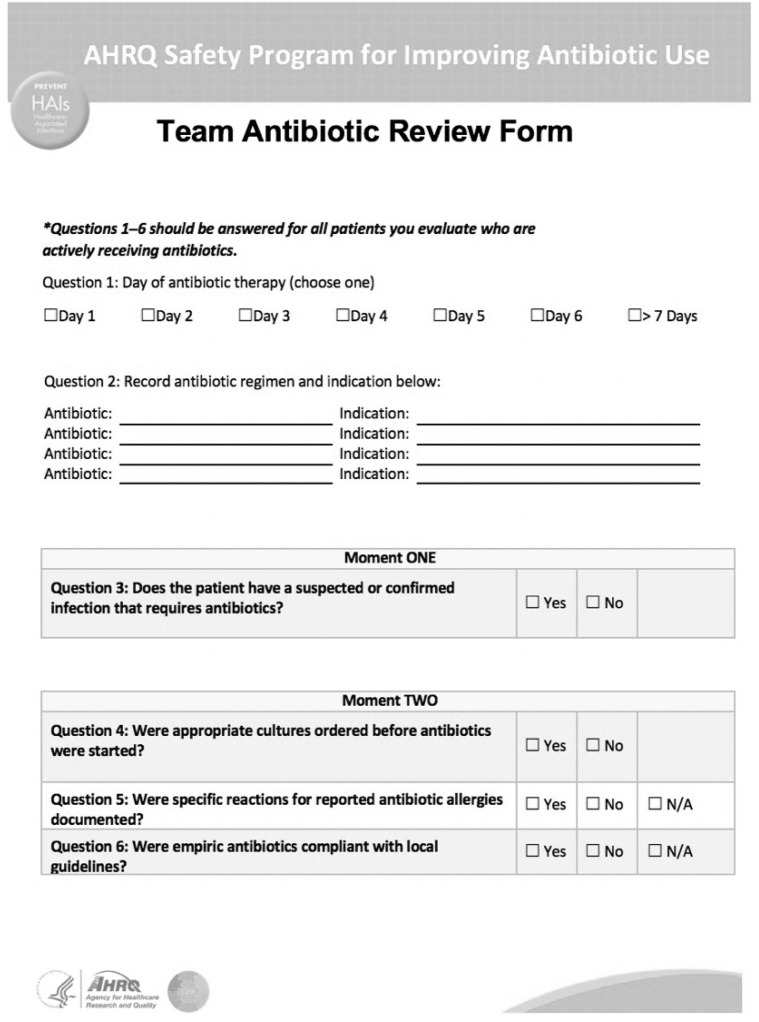

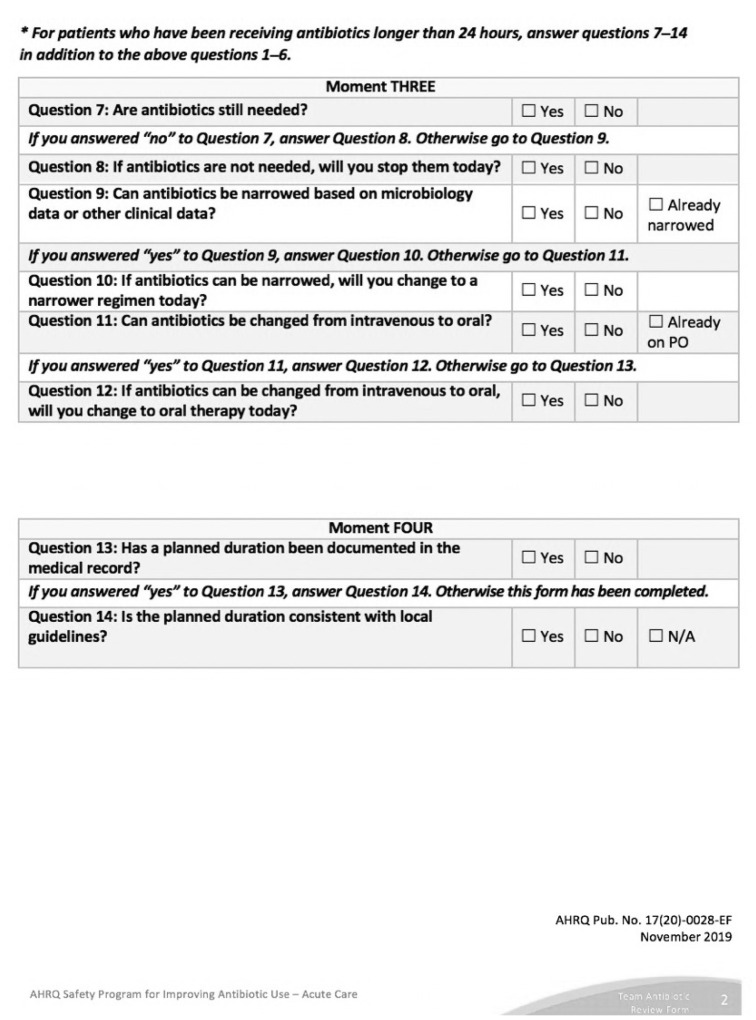
Agency for Healthcare Research and Quality (AHRQ) Team Antibiotic Review Form.

The Agency for Healthcare Research and Quality (AHRQ) Safety Program for Improving Antibiotic Use developed a Team Antibiotic Review Form (TARF) to assist frontline staff in AS practices. The TARF focuses on four key moments in antibiotic prescribing, and consists of 13 questions. A complete TARF can be found in Figure 1 [7]. We hypothesize that the use of a TARF by APPE students will provide a structured method for ASP, resulting in improved student confidence in conducting AS and substantial AS interventions. The objectives of this study were to determine the utility of a structured method of AS by APPE students and assess student perceptions of the tool.

## 2. Methods

Pharmacy students on APPE rotations at a 545 bed, acute care, public New York City hospital were trained to utilize TARFs to contribute to AS between January 2019 and March 2020. Students were assigned to perform AS on a medical intensive care unit with 10 patients or a general internal medicine floor with approximately 30–35 patients. Although a staff pharmacist is assigned to the units on a daily basis for order verification, they are not responsible for systematically performing daily antibiotic reviews. Two faculty preceptors trained students on TARF use during rotation orientation and provided guided feedback throughout the rotation. During orientation, preceptors reviewed each TARF question as it applied to the specific patient so students understood where to locate information in the electronic medical record (EMR) to answer the questions. Additionally, students participated in interdisciplinary team rounds on their assigned units where they could discuss cases with their team and obtain information regarding antibiotic therapy. Students referred to drug information references and hospital policies for dosing and dose-adjustment-related assessment questions, and were responsible for completing TARFs for all patients receiving antibiotic therapy on their assigned unit for the duration of their four-week rotation. All patient TARFs were reviewed with the preceptors, and stewardship interventions were documented in the EMR by preceptors. Once trained, each TARF required approximately 5 min to complete.

At the end of their rotation, students completed an anonymous evaluation regarding their perceptions of using TARFs for AS. The evaluation was created by the faculty preceptors, and evaluation responses were measured using a Likert scale with responses ranging between strongly disagree, disagree, neutral, agree, and strongly agree. The following items were included on the student evaluation:

1. I was confident in evaluating patient antibiotics before my rotation.
2. The TARF helped me evaluate patient antibiotics in a way that was easy to follow.
3. The TARF provided me with an organized and structured way to systematically evaluate antibiotics.
4. The TARF increased my knowledge on how to evaluate patient antibiotics.
5. I feel confident in participating in antimicrobial stewardship after the use of the TARF.
6. My knowledge of antimicrobial stewardship increased after my rotation.
7. I used the information and skills I learned about antimicrobial stewardship after my rotation (for example, I used the skills or performed antimicrobial stewardship during another rotation).

Preceptors quantified total APPE student AS interventions during the study period. Data analysis was conducted using descriptive statistics. The Fisher’s Exact Test was used to compare student’s confidence before and after using TARFs. 

## 3. Results

Twenty-six APPE students participated in AS using TARFs from January 2019 to March 2020. Twenty-three of the students (88.4%) completed a post-rotation evaluation of the TARF. Students included were at various points in their APPE year, with the majority (82.6%) having at least two prior APPE rotations. Approximately 92% of these students had not performed AS activities prior to this rotation experience.

**Figure 2:**
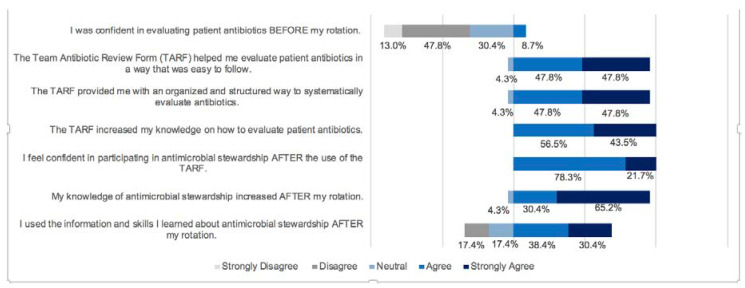
Student Evaluation Responses.

Figure 2 shows that only 8.7% of students reported feeling confident in evaluating patient antibiotics before using the TARF. Nearly 96% of students agreed or strongly agreed that using TARFs helped them evaluate patient antibiotics in a way that was easy to follow. Additionally, about 96% reported that the TARF provided them with an organized and structured way to systematically evaluate antibiotics. All students felt that the TARFs increased their knowledge on how to evaluate patient antibiotics. About 65% of students reported using the information and skills learned about AS through the TARF after completing their rotation, with 34.8% agreeing and 30.4% strongly agreeing. Significantly more students were confident in participating in antimicrobial stewardship after using the TARF (100% vs. 8.7%, *p* < 0.00001).

In total, APPE students were responsible for 889 interventions using TARFs. As seen in Table 1, the most common intervention types by students were prospective audit and feedback (57.4%), therapeutic drug monitoring (19.6%), antibiotic de-escalation (8.5%), and therapy/dosing adjustment recommendations (7.8%). The remaining intervention types included duration of therapy recommendations (4.8%) and intravenous to oral conversion (1.5%). The majority of interventions by APPE students (99%) arose from direct use of the TARF and aligned with the specific questions in the TARF. 

APPE students’ interventions accounted for approximately 54% of total pharmacy AS interventions. Types of interventions made by pharmacy students were similar to those made by the hospital’s stewardship pharmacist. Pharmacy students more commonly made interventions on narrower spectrum antibiotics, whereas the focus of the stewardship pharmacists’ interventions was on broad spectrum agents such as meropenem, vancomycin, and piperacillin/tazobactam.

## 4. Discussion

The use of TARFs allowed APPE students to provide substantial contributions to AS at our institution, where pharmacist involvement in stewardship is hindered by resource availability. Incorporating pharmacy students helped to expand the number of antimicrobials that could be reviewed on a daily basis. Prior to involvement of APPE students, faculty preceptors were not involved in AS. Only one pharmacist was able to participate in AS services as a piloted service in a limited capacity. TARF use also provided a means for teams to conduct antibiotic timeouts, as APPE students frequently completed TARFs during interdisciplinary rounds. This process of involving APPE students in our ASP aligns with ASHP’s position statement on Pharmacist’s Role in Antimicrobial Stewardship and Infection Prevention and Control by providing AS exposure to students and expanding pharmacy education to help develop qualified pharmacists [3].

Additionally, TARFs helped students build their knowledge and confidence in performing AS activities. Prior studies indicate that while pharmacy students perceive ASP to be important, they desire more knowledge on the topic [8]. TARFs helped students to identify and organize information related to antibiotics and apply their findings. In the study by Benson, APPE students were incorporated in the ASP at a long-term acute care hospital. Students received training and used a standardized form to guide their data collection and monitoring of all infection-related patient problems. The student responsibilities included optimizing antimicrobial dosing, serum drug concentration monitoring, duration of therapy, and more, which closely resembled the components of the TARF structured evaluation form used by our APPE students. Benson measured the impact of ASP by reporting the change in their antimicrobial acquisition cost two years after implementing APPE involvement in their ASP. The mean antimicrobial costs per patient day were found to be $75.37 ± $11.85 at baseline and decreased to $64.13 ± $13.78 with APPE involvement, a 14.9% decrease that was calculated to be a cost savings of $261,630 over two years. However, this study did not quantify the number of interventions made by APPE students, nor did it report a student evaluation of the standardized form used for their monitoring responsibilities. Similar to our study, Benson found that pharmacy students played an important role in ASP at their resource limited hospital [5].

In another study, Laible and colleagues looked at the implementation of a pharmacist-led stewardship program composed of a clinical pharmacist, infectious disease physicians, APPE students, and PGY-1 and PGY-2 pharmacy residents. All participated in prospective audit and feedback, dose optimization, and antibiotic streamlining as targeted recommendations. The most accepted type of recommendation was for the discontinuation of antimicrobials [6]. With the comprehensive review process outlined by the TARF, APPE students were able to identify seven intervention types, resulting in 889 interventions over a shorter time frame, suggesting that TARF use allows for broader application than having students carry out select intervention types. 

Limitations of our study included that over the study time frame, only a small group of students (26 students) had APPE rotations at the hospital and were therefore trained on the TARF for AS. Evaluations of the TARF represents the thoughts and opinions of a small percent of students. Due to unforeseen circumstances or periods of time when preceptors were not assigned APPE students, AS services, TARF use, and interventions from APPE students decreased. Future research may include evaluation of student acceptance rate of the TARFs and acceptance of student interventions by medical teams. Evaluation of interventions against standardized AS metrics will help further define the role of APPE students in ASPs.

## 5. Conclusions

Data from the anonymous, voluntary student evaluation regarding their experiences and opinions of the TARF for AS while on rotation suggest that utilization of the tool was beneficial for student learning and participation. This structured method provides a guide for students who do not have experience with AS to confidently assess patient antibiotics. Student involvement in AS can provide considerable contributions quantified by interventions. For institutions that have limited ASPs, students may prove to be valuable assets to AS. In the future, students may be able to participate in additional stewardship endeavors with implementation of other structured tools.

## Figures and Tables

**Table 1:** Pharmacy Student Interventions (N = 889, % (n)).

Intervention Type	Quantity
Prospective audit and feedback	57.4 (510)
Therapeutic Drug Monitoring	19.6 (174)
Antibiotic de-escalation	8.5 (76)
Therapy/dosing adjustment	7.8 (69)
Duration of therapy	4.8 (43)
IV to PO conversion	1.5 (13)
Prevention of drug-drug interaction	0.4 (4)
